# Metabolomic Analysis Uncovers the Presence of Pimarenyl Cation-Derived Diterpenes as Insecticidal Constituents of *Sphagneticola trilobata*

**DOI:** 10.3390/plants14142219

**Published:** 2025-07-17

**Authors:** Lilia Chérigo, Juan Fernández, Ramy Martínez, Sergio Martínez-Luis

**Affiliations:** 1 Universidad de Panamá, Facultad de Ciencias Naturales, Exactas y Tecnología, Ciudad de Panamá, Panamá 0824, Panama; lilia.cherigo@up.ac.pa (L.C.); ramyjhasser522@gmail.com (R.M.); 2 Centro de Biodiversidad y Descubrimiento de Drogas, Instituto de Investigaciones Científicas y Servicios de Alta Tecnología (INDICASAT AIP), Edificio 208, Ciudad del Saber, Panamá 0843-01103, Panama; juanor_fer11@hotmail.com

**Keywords:** *Sphagneticola trilobata*, *Aphis gossypii*, insecticidal activity, metabolomic analysis, diterpenes

## Abstract

*Aphis gossypii* is a significant global pest that impacts numerous agricultural crops and vegetables, causing direct damage to food plants and indirect damage through the transmission of phytopathogenic viruses, primarily begomoviruses. In Panama, particularly in the Azuero region, viral infections transmitted by this aphid can affect a substantial share of tomato crops cultivated for industrial use. A traditional alternative to synthetic pesticides involves exploring plant extracts with insecticidal properties derived from wild plants found in our tropical forests, which can be easily prepared and applied by farmers. In this context, the present research aimed to evaluate the insecticidal activity of ethanolic extracts from the stems and leaves of *Sphagneticola trilobata* on both nymphs and adults of *A. gossypii*. Mortality was assessed at 24, 48, and 72 h after applying three doses of each extract (25, 50, and 100 µg/L). A standard phytochemical analysis to determine insecticidal activity revealed that both extracts exhibited significant efficacy at the highest concentration tested; however, the leaf extract demonstrated greater effectiveness at lower concentrations. A comprehensive metabolomic study indicated that the active compounds are diterpenes derived from the pimarenyl cation. These compounds have been extensively documented for their insecticidal potential against various insect species, suggesting that ethanolic extracts from this plant could serve as viable candidates for agricultural insecticides to combat aphid infestations.

## 1. Introduction

*Sphagneticola trilobata* is an herbaceous plant from the Asteraceae family, popularly known as Botoncillo in Panama. It is native to neotropical regions and can easily grow in various soil types. As a result, it has become an invasive plant in many countries around the world. *S. trilobata* has a long history of use in traditional medicine across different countries [1,2]. Studies have demonstrated its effectiveness in treating conditions such as rheumatism, back pain, muscle cramps, persistent wounds, stiffness, swelling, arthritic pain, and as an anti-inflammatory and antinociceptive agent. It has also shown activity against fungi, Gram-positive and Gram-negative bacteria, as well as flagellated protozoa [1,2].

Despite its medicinal applications, *S. trilobata* has become a problematic plant in many countries where it has been introduced. It is highly competitive and able to outcompete native plants by more efficiently capturing nutrients, light, and water. Moreover, *S. trilobata* is a fast-growing weed with a widespread distribution, often forming monocultures in the wild or plantations [1,3]. This dual nature positions *S. trilobata* as both a valuable medicinal plant and a problematic invasive species. However, some scientists have sought to find ways to turn its unfavorable properties into a beneficial solution for agricultural challenges. In recent years, there has been a growing interest in using plant-based insecticides as an alternative to synthetic pesticides, which can have negative environmental impacts [4,5,6].

In this context, researchers have explored the potential insecticidal properties of *S. trilobata*. The goal is to investigate whether extracts from this plant could be developed into effective, nature-based insecticides that could help reduce reliance on synthetic chemicals in agriculture. A study conducted in Indonesia found that crude extracts from *S. trilobata* leaves affected the growth and development of *Spodoptera litura* larvae. This polyphagous pest damages many crops, although it does not impact the length and weight of the resulting pupae. Further research analyzing leaf and flower extracts revealed that flower extracts (LC_50_ 277.5 mL/L) were more toxic to *S. litura* larvae than leaf extracts (LC_50_ 373.8 mL/L) [7]. In another study, a 5% *S. trilobata* flower extract exhibited antifeedant activity against *Spodoptera frugiperda*, a new pest that attacks maize plantations in Indonesia. The analyzed extract showed a 66.11% inhibition of feeding activity and resulted in an average larval weight of 0.048 g, which was lower than the control treatment [8].

Inspired by these findings, our research group decided to test the extracts from the leaves and stems of the *S. trilobata* variety found in Panama to evaluate its potential insecticidal properties against *A. gossypii*, a pest that significantly affects the vegetables in our country. *A. gossypii* typically feeds on plant sap and has a wide distribution, affecting numerous cultivated plant species, particularly those in the Cucurbitaceae, Rutaceae, and Malvaceae families. Both adults and nymphs feed on the underside of leaves and tender shoots, extracting plant juices. This feeding can lead to chlorosis in the foliage and may eventually cause premature plant death. Additionally, they often induce leaf deformation, resulting in reduced photosynthetic efficiency. The aphids excrete a honeydew that can promote the growth of sooty mold, leading to product depreciation. Furthermore, these aphids can act as vectors for various viruses that cause plant diseases [9,10].

## 2. Results

### 2.1. Insecticidal Activity

**Figure 1:**
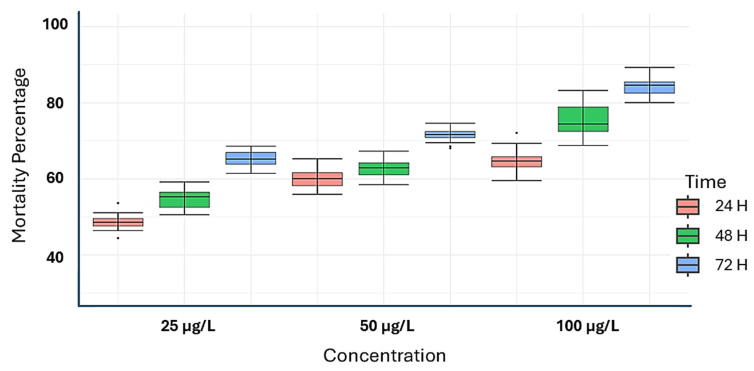
Insecticidal activity of *Sphagneticola trilobata* leaf extract.

**Figure 2:**
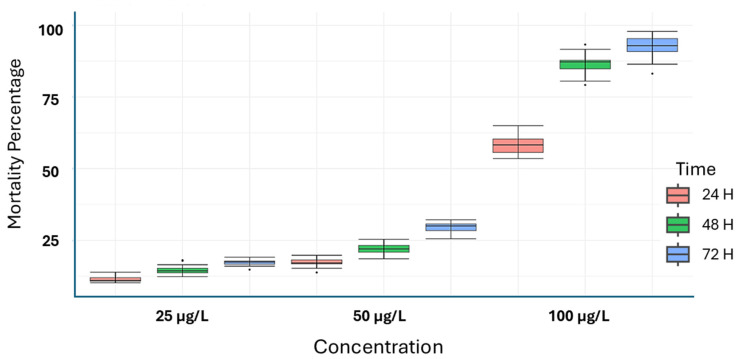
Insecticidal activity of *Sphagneticola trilobata* stem extract.

The organic extract was applied, and the mortality rate was assessed every 24 h over 72 h. Overall, the results indicated that the leaf extract (LD_50_ = 12.70 μg/L) resulted in a significantly higher mortality rate compared to the stem extract (LD_50_ = 63.65 μg/L) and the control treatment by the end of 72 h. Notably, the leaf extract achieved the highest mortality, killing 89% of the insects at 48 h and reaching 92.6% by 72 h—significant figures for a crude extract. In general, the mortality percentage was significantly influenced by several factors, including the plant organ used for the extract, its concentration, and the duration of exposure (See Figure 1 and Figure 2).

### 2.2. GC-MS-Based Metabolomic Analysis

**Figure 3:**
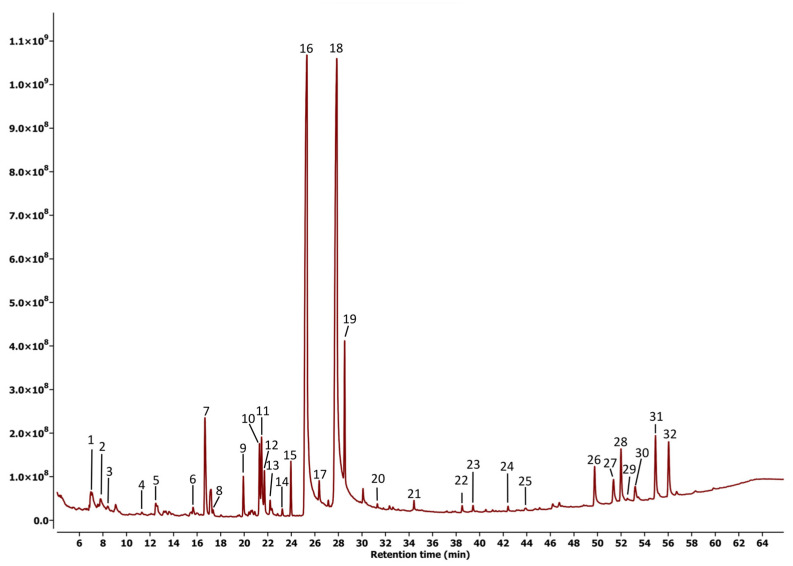
GC-MS chromatogram for the leaf extract of *Sphagneticola trilobata*.

**Figure 4:**
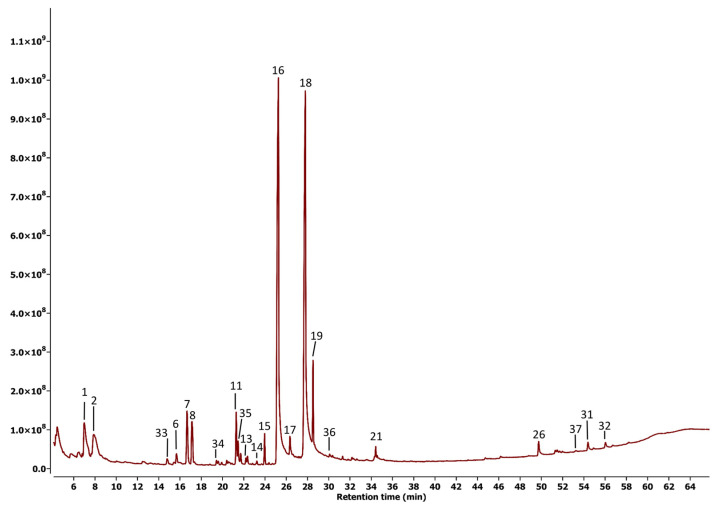
GC-MS chromatogram for the stem extract of *Sphagneticola trilobata*.

Figure 3 and Figure 4 show the chromatograms obtained under the best conditions evaluated during their analysis. These chromatograms reveal a clear difference in the fragment between 50 and 58 min, suggesting that metabolites in this range may be primarily responsible for the observed differences in biological activity.

Table 1 and Table 2 provide a list of compounds identified with a matching percentage of 80% or higher in the NIST20 database. In the case of the leaf extract, 32 compounds were identified, notably including well-characterized groups such as sesquiterpene lactones, ent-kaurane diterpenes, steroids, and triterpenes. In contrast, only 22 compounds were detected in the stem extract, indicating that most of the compounds found in the stem extract were also present in this fraction.

### 2.3. Molecular Networking

**Figure 5:**
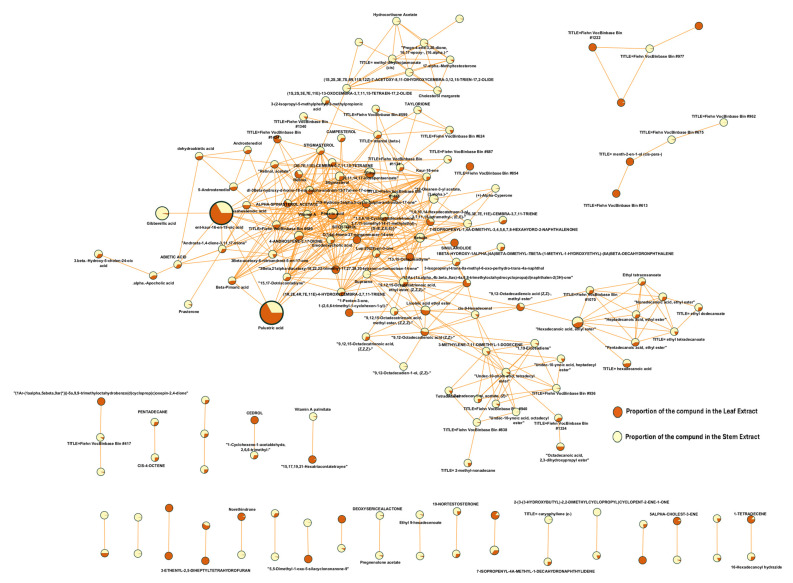
Molecular networks filtered by the relative abundance of ions in the leaf and stem extracts of *Sphagneticola trilobata*. The node size represents the relative abundance of ions. The composition of compounds in the leaf extract is indicated in brown, while the composition in the stem extract is depicted in pale brown.

Figure 5 shows the molecular network generated using the compounds produced by *S. trilobata* leaf extract on the GNPS platform; it can be observed that these compounds are grouped according to their similar chemical characteristics. Each cluster represents a specific group of secondary metabolites produced by this plant, highlighting the presence of triterpenes, sterols, and fatty acids. It is important to note that numerous unique or unidentified compounds do not match known structures or are absent from the database. These nodes may represent new or rare natural compounds or compounds that have not yet been included in the databases utilized by the screening tool. Additionally, the presence of these nodes emphasizes the importance of further studying *S. trilobata* extracts to obtain these compounds, as this could potentially lead to the isolation of new bioactive substances in *S. trilobata*.

**Figure 6:**
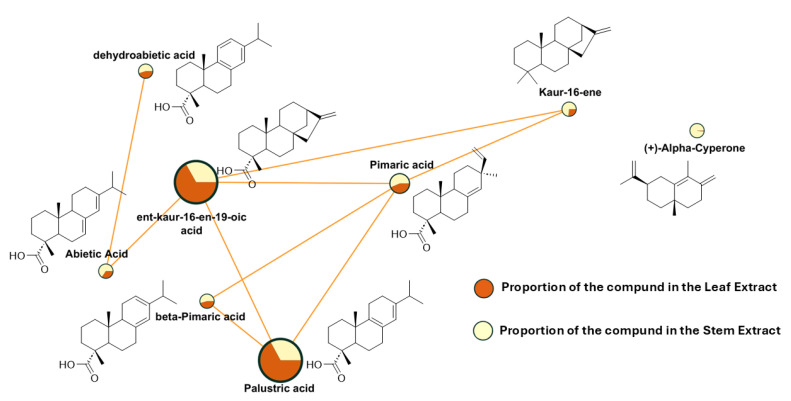
Molecular networks filtered by the relative abundance of ions in the leaf and stem extracts of *Sphagneticola trilobata*. Expansion of the cluster containing compounds previously reported in the literature to exhibit insecticidal activity.

Figure 6 provides a closer view of the clusters containing compounds that have been documented in the literature for their significant insecticidal activity; to enhance visualization, unrelated compounds have been removed. It is important to note that other compounds within the same cluster may also exhibit insecticidal activity, which has yet to be reported. Therefore, verifying their potential will be a challenge for future research.

**Figure 7:**
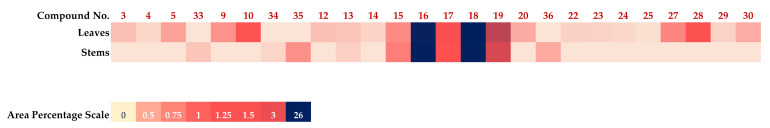
Heatmap displaying the relative concentrations of similar or differential compounds (based on percentage of peak area) in the leaf and stem extracts of *Sphagneticola trilobata*. The compound numbers are indicated at the top of the heatmap, while the specific names of each compound can be found in Table 1 and Table 2.

To visually elucidate the differences in concentrations and the various compounds present in both leaf and stem extracts, a heatmap was generated to compare the relative concentrations of compounds in each organic extract. For this, each extract was injected at the same concentration and analyzed under identical gas chromatography conditions. Figure 7 illustrates the resulting heatmap, clearly highlighting the presence and relative amounts of the compounds that differentiate each extract.

**Figure 8:**
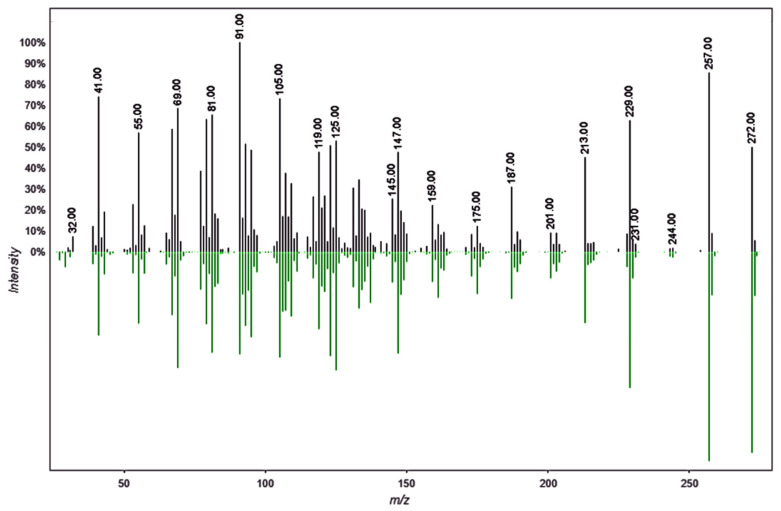
Comparison of the experimentally obtained spectrum of the compound with a retention time of 23.23 min. and the spectrum deposited on the GNPS platform for the compound Kaur-16-ene. The high peak correlation confirms the compound’s identity with a high degree of certainty. In black is the spectrum obtained experimentally, in green the reference spectrum.

To ensure a high degree of certainty and confidence in the results, we report only those compounds that exhibited a high percentage of similarity with the reference spectra from the databases used in the analysis. This approach provides a robust basis for confirming their identities. For many years, the electron impact ionization (EI) technique has been utilized to identify compounds based on their fragmentation patterns, which serve as a unique fingerprint for each compound. Consequently, a higher percentage of similarity (NIST20) or cosine factor (GNPS) indicates greater certainty in the identification of the compounds. Figure 8 illustrates the compound identification process conducted on the GNPS platform in the present study. In this figure, the platform compares the spectrum obtained for the compound Kaur-16-ene (with a retention time of 23.23 min in the leaf extract) against a reference spectrum for the same compound stored in the database, generating a cosine factor of 0.94 based on the number of shared fragments.

## 3. Discussion

Globally, aphids significantly impact crops such as zucchini, melon, cucumber, eggplant, strawberries, cotton, and citrus [9,10]. In the Azuero region of the Republic of Panama, they are particularly problematic as common pests of tomato crops, resulting in substantial economic losses for farmers. Therefore, it is crucial to explore new pest control alternatives, especially sustainable solutions that are ecologically friendly and less toxic to farmers, to align with global trends toward sustainability. In this context, this research proposal aims to explore the potential insecticidal properties of *S. trilobata*. Specifically, the primary objective is to investigate whether extracts from this plant can be developed into effective natural insecticides, thereby reducing dependence on synthetic chemicals in agriculture.

As shown in Figure 1 and Figure 2, both *S. trilobata* extracts demonstrate significant insecticidal activity, achieving up to 80 percent insect inhibition at the highest concentration tested. This finding raises optimism for the potential practical application of these extracts, pending further optimization studies. A detailed analysis reveals distinct differences in the effects of each extract. The leaf extract shows a pronounced insecticidal effect even at lower concentrations, while the stem extract exhibits potential activity only at the highest concentration. This suggests that the active components may differ between the extracts or that factors such as solubility could be influencing their biological activity.

The insecticidal activity of the leaf extract is superior, demonstrating a greater inhibitory effect at lower concentrations; however, the activity becomes quite similar at the highest concentration tested. This finding is significant for future optimization studies. It is important to note that the current study was conducted under laboratory conditions, which may not directly translate to agricultural systems. Nevertheless, if these results are consistent, the *S. trilobata* leaf extract would undoubtedly be a more favorable option, as emerging trends emphasize the use of effective products at lower concentrations [11].

To gain a clearer understanding of the differences in metabolic composition, a comprehensive comparative metabolic analysis was conducted. To achieve this, a conventional metabolomic analysis was conducted alongside an analysis using the GNPS (Global Natural Products Social) platform. In recent years, GNPS has made significant and transformative contributions to the field of metabolomics, particularly in the analysis of bioactive compounds in natural extracts or essential oils [12]. Electron impact ionization is a fundamental tool for identifying compounds, as it generates fragmented ions in a specific manner for each compound. This results in a unique fragmentation pattern that facilitates identification through the NIST database in classical metabolomics or using the GNPS platform by applying a modern metabolomic approach. On the other hand, the GNPS platform, through the Mashub algorithm for gas chromatography (GC), improves the compound identification process by performing auto deconvolution of compound fragmentation patterns applying an unsupervised non-negative matrix factorization. This approach also quantifies the reproducibility of fragmentation patterns in the analyzed samples. Additionally, Mashub automatically optimizes parameters by applying fast Fourier transform, multiplication, and inverse Fourier transform for each ion in complete datasets, followed by unsupervised non-negative matrix factorization (a one-layer neural network) [13]. This results in an orthogonal metric of the deconvoluted spectral quality. Subsequently, the spectra generated by GNPS-MSHub are searched and compared with spectra in libraries. Matches are assessed using criteria such as the number of matched ions, Kovats index, balance score, cosine score, and abundance. As a result, GNPS provides all candidate matches that meet the user-defined filtering criteria. By applying a balance score of 65%, the platform ensures that only high-quality deconvoluted spectra are compared to the reference library, leading to more accurate and precise detections. This is particularly significant for certainty and accuracy in compound identification, considering that electron impact ionization (70 eV) has been used for decades, resulting in an accumulation of approximately 1.2 million reference spectra [13].

Our study was focused on the analysis of volatile metabolites, as it has been demonstrated that these kinds of metabolites play a key role in the natural defense response of plants against their predators, including insects, pathogenic microorganisms, and other pests [14,15]. The results (Figure 3 and Figure 4, Table 1 and Table 2) revealed a distinct variation in metabolic profiles, particularly regarding the quantity and nature of the compounds present. The significant difference in the chemical composition of the two extracts provides reliable evidence that the observed variation in biological activity is related to the presence of primary active components of differing natures.

The leaf extract contains several diterpenes. Given the literature indicating the strong insecticidal activity of these compounds [16,17,18]. They likely contribute to the inhibition of *Aphis gossypii* observed in both extracts, particularly in the leaf extract. Although the stem extract also contains these diterpenes, it lacks several apolar compounds present in the leaf extract, as well as other polar compounds that were not detected by gas chromatography-mass spectrometry (GC-MS). These differences significantly influence the insecticidal activity, likely explaining why the stem extract is nearly inactive at low concentrations but effective at higher concentrations. This further suggests that the overall potency of both extracts results from a synergistic effect driven by the concentration of each compound within the extract and the total number of active compounds present. Furthermore, it is also highly likely that active compounds in the stem extract are either not yet described in the literature or not included in the databases used for GC-MS analysis.

An important consideration is that during the assay, the stem extract may have encountered solubility issues with its active components, as the lowest concentrations tested did not exhibit significant activity. Additionally, the behavior of the analyzed concentrations (25, 50, and 100 mg/mL) does not align with the fundamental principle of the concentration–response relationship, which states that the magnitude of the effect typically increases proportionally with concentration. Although this linear relationship is generally observed across various concentrations, it is not universally maintained. Biological responses often follow a sigmoid curve, where the initial response is linear, stabilizes, and eventually reaches a plateau, indicating that higher concentrations do not yield any additional effect [19]. Solubility issues may also have impacted the metabolomic analysis, as it is likely that the active components in the stems are polar in nature and, therefore, do not possess sufficient volatility for detection by GC-MS. This limitation is relevant to our research group’s expertise, which may explain why the primary metabolites of this extract are not reported in the present manuscript. Notably, our report reveals a clear difference in the composition of volatile metabolites between the two extracts, with a greater quantity of compounds found in the leaf extract. Additionally, Figure 4 illustrates two nodes in the central part of the network, highlighting compounds that are predominantly present in the stem extract.

The principal compounds with insecticidal properties detected in *S. trilobata* are diterpenes derived from the pimarenyl cation (Figure 6). These compounds play a crucial role in the defense of conifers against predatory insects, such as bark beetles. Bark beetles initiate attacks on living trees, overcoming host defenses through mass assaults and relying on their symbiotic fungi to kill the tree via toxin production and the growth of conductive elements. Conifers differ in their reliance on constitutive and inducible defenses; for instance, pines store large amounts of oleoresin, while firs produce oleoresin in response to injuries [16,17,18]. Some plant oleoresins contain significant amounts of diterpenes derived from pimarenyl cation [16,17,18,20], which studies have shown to influence the feeding behavior of pearl cutworm larvae by acting as deterrents. In one study, diet choice tests were conducted to demonstrate that second instar larvae actively selected diets with lower levels of resin acids when presented with various diets randomly arranged in a Petri dish. The number of larvae feeding on the diets increased as the concentration of resin acids decreased, indicating a significant negative correlation between resin acid levels and larval feeding activity. This suggests that higher concentrations of resin acids in the diet deter larval feeding, likely contributing to the overall growth inhibition observed in the bioassays. Specifically, abietic acid, dehydroabietic acid, and isopimaric acid demonstrated strong antifeedant activity against variegated cutworm larvae. The authors tested both a natural mixture of resin acids and pure commercial compounds, finding that both the individual acids and the natural mixtures significantly inhibited larval growth and feeding. The results indicated that each resin acid exhibited a dose-dependent inhibitory effect, with no apparent synergistic interactions enhancing their activity. Notably, this study indicates that individual resin acids with a pimaric acid backbone exhibit significant insecticidal activities due to their antifeedant properties, suggesting their crucial role in the bioactivity of tall oil against the cutworm in this specific context [21].

**Figure 9:**
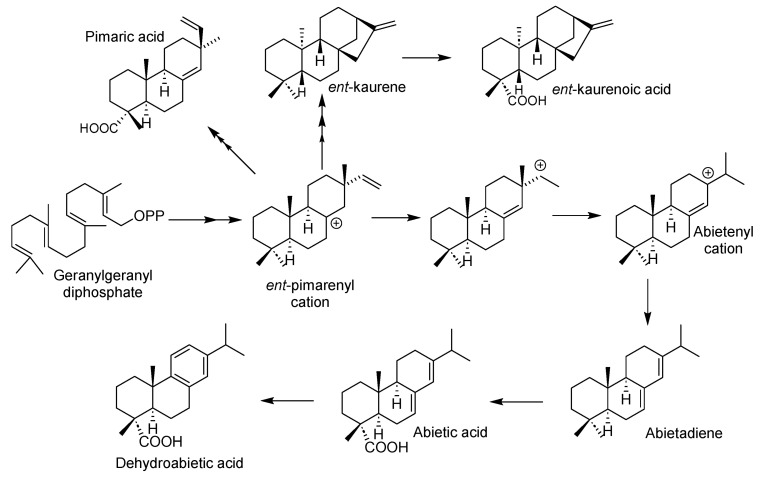
Pathway overview for diterpene biosynthesis in *Sphagneticola trilobata*.

It is important to note that six of the diterpenes identified as responsible for the insecticidal activity share the same biogenetic origin (Figure 9). This observation reinforces the presence of such compounds in the extract of *S. trilobata* because it is well documented that plants typically produce a variety of compounds belonging to the same chemical families rather than isolated compounds.

In general terms, plant oleoresins are primarily composed of terpene-type compounds, which are biosynthetically derived from isopentenyl diphosphate via two pathways: the acetate-mevalonate pathway in the cytosol for sesquiterpenes and triterpenes, and the pyruvate-glyceraldehyde-3-phosphate pathway in plastids for monoterpenes, diterpenes, and tetraterpenes. Well-documented studies in conifers have revealed that each type of terpene plays a vital role in plant defense [1,2]. The turpentine fraction, containing monoterpenes and sesquiterpenes, includes compounds toxic to bark beetles and microbial invaders, impeding weevil predation and microbial growth following injury. The resin subsequently acts as a physical barrier; upon exposure to the atmosphere, volatile turpentine evaporates, leaving a hardened mass of diterpenoid resin acids that seal the wound, effectively trapping or preventing the movement of invading weevils and pathogenic fungi within the tree’s tissues [1,2]. The presence of various types of terpenes in the analyzed extracts suggests that this defensive mechanism, well documented in conifers, is likely also present in *S. trilobata*. Given that plants in tropical environments are often exposed to a variety of insect pests, they require robust natural defense mechanisms for their survival. 

In summary, all the experimental evidence presented and the information previously published and discussed above indicate that the organic extracts of *S. trilobata* possess significant insecticidal potential. However, it is important to note that future studies are required, which should include isolation, structural characterization of the active compounds (by NMR), and characterization of the biological activity in vivo, as well as experiments to determine the molecular mechanism of action of the active compounds.

## 4. Materials and Methods

### 4.1. Plant Material

Permission for the plant collection was obtained from the country’s Ministry of the Environment of Panama (MiAmbiente, permit ARGB-015-2021). *S. trilobata* (Asteraceae) was collected by Ramy Martinez in February 2021 in Cerro Azul, province of Panama, Republic of Panama. The plant was identified for comparison with an *S. trilobata* previously deposited in the herbarium of the University of Panama and using the work reported by Wald 2017 [22,23]. The plant’s aerial parts (leaves and stems) were carefully collected, placed in plastic bags, and transported to the laboratory for subsequent extract preparation. During the plant collection process, great care was taken to select organs free of disease and without apparent insect damage. The plant species’ type material was identified and entrusted to the Laboratory of the Vice-Presidency of Research and Postgraduate Studies at the University of Panama, where it was deposited for future reference.

### 4.2. Preparation of Extracts

The collected plant material was washed to remove sand, dust, and other contaminants, and then dried at room temperature for 5–6 days in a clean environment. The stems were separated from the leaves after drying in the shade and then crushed. The crushed plant material was extracted using a maceration process. For this, 500 g of plant material (from each plant organ) was placed in Erlenmeyer flasks and completely covered with 99% ethanol. The mixture was left to macerate for 48 to 72 h in a cool place protected from light. Afterward, the liquid was filtered and concentrated under a vacuum in a rotary evaporator until dryness was reached. The dried extract was placed in a sterile vial and stored at 5 °C until the bioassay was performed. For the ethanol extract bioassay, 10 µL samples containing 100 µg/L, 50 µg/L, and 25 µg/L of the crude extract were taken [24,25].

### 4.3. Bioassay

The bioassay in this study was conducted using adults and fourth-instar nymphs of *Aphis gossipii*, following the residual toxicity (leaf-dip) method described by Ahmed et al. (2020) with modifications [26]. Aphid colonies were originally collected from agricultural fields in Villa Lucre, Panama City, and were subsequently maintained under laboratory conditions on *Catharanthus roseus* plants without prior insecticide exposure. Colonies were acclimated in a controlled environment chamber at 28 ± 4 °C, 70 ± 5% relative humidity, and a 12:12 h light/dark photoperiod for 48 h prior to the bioassay. For each treatment, *C. roseus* leaves approximately 5 cm in diameter were immersed for 10 s in ethanolic extract solutions prepared at concentrations of 25, 50, or 100 µg/L. This immersion duration was selected based on a previously reported standardized protocol [26], which has been validated for achieving consistent residue coverage without damaging the leaf tissue. Treated leaves were placed in 6 cm diameter Petri dishes containing a 2% agar layer to maintain hydration and were left at room temperature for 24 h to allow solvent evaporation and avoid excess moisture, which could adversely affect aphid survival. Ten aphids were carefully transferred onto each treated leaf using a fine camel-hair brush, ensuring no injury during handling. Aphids were allowed to feed freely throughout the exposure period. Treatments were randomly assigned to Petri dishes, and mortality assessments were conducted by an independent observer blinded to treatment identity. Each concentration and control were replicated in three independent Petri dishes containing 10 aphids each, and the experiment was repeated once under identical conditions to confirm reproducibility, resulting in six replicates per treatment overall. The negative control consisted of a solution prepared with 9 mL of distilled water and 1 mL of 99% (*v*/*v*) ethanol. The positive control was prepared with Imidacloprid^®^ at a final concentration of 0.04 mg/L (0.02 mg dissolved in 500 mL distilled water), corresponding to field-recommended application rates for aphid management. Mortality was assessed at 24, 48, and 72 h post-treatment. Individuals were recorded as dead when no movement was observed after gentle prodding with a fine brush. Percentage mortality was calculated using the following formula:

$$Mortality\left(\%\right)=\left(\frac{Number\;of\;dead\;aphids}{Total\;number\;of\;aphids}\right)\times 100$$

The results obtained from the bioassay were presented as percentage mortality. The statistical analysis was conducted using the R program [27], and the LC_50_ value was calculated using the R program and the package drc [28]. The code used to create the included a dataframe with two columns: dose (exposure levels: 25, 50, and 100 μg/L) and mortality (proportion of deaths expressed as a percentage of the average calculated from at least 20 individuals). The boxplot () function was applied using the formula Mortality~Dose to create a boxplot that groups the response (mortality) by the different doses. Additionally, customization parameters for axes, colors, and borders are specified to enhance the visualization.

### 4.4. GC-MS-Based Metabolomic Analysis of the S. trilobata Extract

The untargeted metabolomic analysis of the ethanol fraction from *S. trilobata* was conducted using an Agilent 8890 gas chromatograph coupled with an Agilent 5977C mass spectrometer. Separation was performed using an HP-5MS capillary column (30 m length, 25 mm ID, 0.25 μm df, Agilent, Santa Clara, CA, USA), with high-purity helium as the carrier gas flowing at a constant rate of 1.1 mL/min. The GC temperature program was initiated at 150 °C, followed by oven temperature ramps of 3 °C/min to 250 °C and 2 °C/min to 305 °C. A final 5-min hold was maintained at 305 °C. The electron impact (EI) ion source was held at 250 °C with a filament bias of −70 V. Full scan mode (m/z 30–600) was employed, and data acquisition was performed at a rate of 20 spectra/second in the MS setting. Agilent Mass Hunter Workstation software (version 10.1.49) was used for data acquisition and processing. The mass spectra corresponding to each peak were searched using the NIST20 spectral library with a retention similarity of 90% or more [25].

### 4.5. Molecular Networking

The GC-EI/MS data were initially processed using the Mass Hunter software from Agilent (version 10.1.49). Mass spectrometry molecular networks were generated using the GNPS platform (http://gnps.ucsd.edu, accessed on 14 July 2025) [13,29]. Since the mass data obtained from the EI experiments did not have pre-selected precursor ions (referred to as the DIA acquisition format), spectral deconvolution was necessary. For this purpose, the GC-MS data were analyzed and processed using the MS Hub algorithm [13].

The raw data were submitted for processing to the spectral network algorithm (GNPS), and these were fitted using the following parameters: a fragment ion mass tolerance of 0.5 Da, a minimum of 5 matched peaks, and a score threshold of 0.7. In terms of search options, the following parameters were used: a gold library class, the top history per spectrum was selected 10, and both the NIST20 and GNPS spectral libraries were utilized. The minimum pair cosine similarity was set to 0.85, and the network topK to 15 in the advanced network options. For more detailed information about the network on GNPS, visit: https://gnps.ucsd.edu/ProteoSAFe/status.jsp?task=1d544c456ffb4bd1ba55a128aaa28294, (accessed on 14 July 2025). The network visualization was performed using Cytoscape v.3.4.3. Node colors and sizes were assigned based on the metadata files, and the thickness of the edges represented the cosine similarity scores, with thicker lines indicating a higher degree of similarity [25].

## 5. Conclusions

Organic ethanolic extracts prepared separately from the leaves and stems of the *Sphagneticola trilobata* plant exhibit inhibitory activity against *Aphis gossypii*, an aphid that causes significant economic losses in tomato crops in Panama and across various crops globally. The leaf extract demonstrated greater in vitro efficacy at low concentrations, while both extracts exhibited similar efficacy at the highest concentration tested. The difference in effectiveness is likely related to the solubility of the components present in each extract, presumably due to a higher concentration of polar components in the stem extract. Metabolomic analysis suggests that diterpene compounds may be among the active components in both extracts. The overall results, combined with the previously reported insecticidal activity attributed to the diterpenes identified in the *S. trilobata* extract, along with earlier studies indicating that extracts of this plant exhibit toxic and antifeedant activity against other insect species, suggest that *S. trilobata* extracts may be promising candidates for the development of natural insecticides against *A. gossypii*.

## Figures and Tables

**Table 1:** Chemical composition of *Sphagneticola trilobata* leaf extract.

No.	Compound	RT (min)	A%	MW (g/mol)	MF	P% or C	L
1	Espatulenol	6.98	1.53	220.35	C_15_H_24_O	98	Nist 20
2	Junenol	7.80	1.07	222.37	C_15_H_26_O	90	Nist 20
3	Aromadendrene	8.41	0.37	204.35	C_15_H_24_	90	Nist 20
4	Ethyl Myristate	11.31	0.15	256.42	C_16_H_32_O_2_	92	Nist 20
5	Neophytadiene	12.49	0.68	278.50	C_20_H_38_	99	Nist 20
6	Palmitic Acid	15.66	0.28	256.42	C_16_H_32_O_2_	99	Nist 20
7	Palmitic acid ethyl ester	16.67	2.82	284.50	C_18_H_36_O_2_	92	Nist 20
8	Manoyl oxide	17.35	0.20	290.50	C_20_H_34_O	97	Nist 20
9	Phytol	19.93	0.83	296.50	C_20_H_40_O	90	Nist 20
10	9,12-Octadecadienoic acid, ethyl ester	21.29	1.46	308.50	C_20_H_36_O_2_	99	Nist 20
11	Linoleic acid ethyl ester	21.47	1.91	308.50	C_20_H_36_O_2_	99	Nist 20
12	8-Methylenedispiro [2.1.2.4] undecane	21.72	0.37	162.27	C_12_H_18_	90	Nist 20
13	Ethyl Stearate	22.20	0.33	312.50	C_20_H_40_O_2_	99	Nist 20
14	Kaur-16-ene	23.23	0.16	272.50	C_20_H_32_	95	Nist 20
15	Kaur-16-en-18-al	23.97	0.91	302.50	C_20_H_30_O_2_	99	Nist 20
16	ent-kaur-16-en-19-oic acid	25.27	26.11	302.40	C_20_H_30_O_2_	0.87	GNPS
17	Dehydroabietic acid	26.37	2.23	300.40	C_20_H_28_O_2_	91	Nist 20
18	Palustric acid	27.85	26.86	302.50	C_20_H_30_O_2_	0.87	GNPS
19	Pimaric acid	28.53	8.14	302.50	C_20_H_30_O_2_	0.88	GNPS
20	Neoabitic acid	31.31	0.58	302.50	C_20_H_30_O_2_	92	Nist 20
21	3 beta-Hydroxy-5-cholen-24-oic acid	34.42	0.35	302.50	C_20_H_30_O_2_	0.82	GNPS
22	Squalene	38.51	0.20	410.70	C_30_H_50_	99	Nist 20
23	Epoxyprogesterone	39.43	0.17	328.40	C_21_H_28_O_3_	0.83	GNPS
24	(1S,2S,3E,7S,8R,11S,12Z)-7-acetoxy-8,11-dihydroxycembra-3,12,15-trien-17,2-olide	42.41	0.13	392.50	C_22_H_32_O6	0.82	GNPS
25	Alpha-sprinasterol acetate	44.74	0.06	454.70	C_31_H_50_O2	0.80	GNPS
26	Stigmasterol	49.76	1.34	412.70	C_29_H_48_O	96	Nist 20
27	beta-Amyrone	51.36	0.96	424.70	C_30_H_48_O	96	Nist 20
28	beta-Amyrin	51.99	1.63	426.70	C_30_H_50_O	90	Nist 20
29	(+)-Alpha-cyperone	52.54	0.17	218.83	C_15_H_22_O	0.79	GNPS
30	alpha-Amyrin	53.20	0.56	426.70	C_30_H_50_O	91	Nist 20
31	beta-Amyrin acetate	54.93	2.01	468.80	C_32_H_52_O_2_	83	Nist 20
32	Friedelanol	56.05	1.71	428.70	C_30_H_52_O	91	Nist 20

RT: Retention time, A%: area percentage, MW: molecular weight, MF: molecular formula, P%: probability percentage, C: cosine factor, L: library.

**Table 2:** Chemical composition of *Sphagneticola trilobata* stem extract.

No.	Compound	RT (min)	A%	MW (g/mol)	MF	P% or C	L
1	Espatulenol	6.97	0.56	220.350	C_15_H_24_O	92	Nist 20
2	Junenol	7.82	0.49	222.370	C_15_H_26_O	91	Nist 20
33	Palmitic acid methyl esther	14.79	0.31	270.450	C_17_H_34_O_2_	99	Nist 20
6	Palmitic Acid	15.67	0.56	256.420	C_16_H_32_O_2_	99	Nist 20
7	Palmitic acid ethyl ester	16.67	2.99	284.500	C_18_H_36_O_2_	95	Nist 20
8	Manoyl oxide	17.15	2.01	290.500	C_20_H_34_O	90	Nist 20
34	Linoleic acid methyl ester	19.44	0.15	294.472	C_19_H_34_O_2_	99	Nist 20
11	Linoleic acid ethyl ester	21.29	1.58	308.500	C_20_H_36_O_2_	99	Nist 20
35	Linolenic acid ethyl ester	21.47	0.86	306.500	C_20_H_34_O_2_	99	Nist 20
13	Ethyl Stearate	22.20	0.22	312.500	C_20_H_40_O_2_	99	Nist 20
14	Kaur-16-ene	23.23	0.15	290.500	C_20_H_34_O	90	Nist 20
15	Kaur-16-en-18-al	23.97	1.03	302.500	C_20_H_30_O_2_	99	Nist 20
16	ent-kaur-16-en-19-oic acid	25.26	26.47	302.400	C_20_H_30_O_2_	0.87	GNPS
17	Dehydroabietic acid	26.35	1.56	300.400	C_20_H_28_O_2_	91	Nist 20
18	Palustric acid	27.79	26.57	302.500	C_20_H_30_O_2_	0.87	GNPS
19	Pimaric acid	28.52	5.4	302.500	C_20_H_30_O_2_	0.88	GNPS
36	Kaur-19-oic acid	30.08	0.6	302.500	C_20_H_30_O_2_	99	Nist 20
21	3 beta-Hydroxy-5-cholen-24-oic acid	34.42	0.3	302.500	C_20_H_30_O_2_	0.82	GNPS
26	Stigmasterol	49.75	0.78	412.700	C_29_H_48_O	0.97	GNPS
37	13,15-Octacosadiyne	53.25	0.09	386.700	C_28_H_50_	0.87	GNPS
31	beta-Amyrin acetate	54.39	0.46	468.800	C_32_H_52_O_2_	83	Nist 20
32	Friedelanol	56.05	0.31	428.700	C_30_H_52_O	91	Nist 20

RT: retention time, A%: area percentage, MW: molecular weight, MF: molecular formula, P%: probability percentage, C: cosine factor, L: library.

## Data Availability

The original contributions presented in the study are included in the article, further inquiries can be directed to the corresponding author.

## Abbreviations

The following abbreviations are used in this manuscript:

NIST20	National Institute of Standards and Technology Database 20
GC-MS	Gas chromatography-mass spectrometry
GNPS	Global Natural Product Social Molecular Networking
